# Hospital acquired COVID-19 infections amongst patients before the rollout of COVID-19 vaccinations, a scoping review

**DOI:** 10.1186/s12879-022-07128-5

**Published:** 2022-02-10

**Authors:** Nobubelo K. Ngandu, Tshiamo M. Mmotsa, Reshmi Dassaye, Alice Thabetha, Willem Odendaal, Natasha Langdown, Duduzile Ndwandwe

**Affiliations:** 1 HIV Prevention Research Unit, South African Medical Research Council, Cape Town, South Africa; 2 Faculty of Health Sciences, University of Cape Town, Cape Town, South Africa; 3 Department of Psychiatry, Stellenbosch University, Cape Town, South Africa; 4 Knowledge and Information Management Services, South African Medical Research Council, Cape Town, South Africa; 5 Cochrane, South African Medical Research Council, Cape Town, South Africa

**Keywords:** COVID-19, Hospital acquired infections, Patients, Public healthcare

## Abstract

**Background:**

Hospital settings are at increased risk of spreading Coronavirus Disease 2019 (COVID-19) infections, hence non-pharmaceutical prevention interventions (NPPIs) and prioritized vaccination of healthcare workers and resident patients are critical. The status of COVID-19 hospital acquired infections (HAIs) in low-income settings is unclear. We aimed to identify and summarize the existing evidence on COVID-19 HAIs amongst patients, prior to the rollout of vaccines in countries worldwide.

**Methods:**

We conducted a scoping review of English peer-reviewed literature in PubMed, Web of Science and Scopus using a combination of selected search terms. Full texts articles presenting results on COVID-19 HAIs in hospitalised patients before the rollout of vaccines in countries worldwide were eligible. Data extracted from eligible articles included estimates of COVID-19 HAIs, country, and type of hospital setting, and was summarized narratively. Quality assessment of included articles was not possible.

**Results:**

Literature searches generated a total of 5920 articles, and 45 were eligible for analysis. Eligible articles were from Europe, North America, Asia, and Brazil and none were from low-income countries. The proportion of COVID-19 HAIs ranged from 0% when strict NPPIs were applied, to 65% otherwise. The estimates of COVID-19 HAIs did not differ by country but were lower in studies conducted after implementation of NPPIs and in specialized hospital settings for operative surgery. Studies conducted before the implementation of NPPIs or in long-term care and psychiatric wards often reported high estimates of HAI. Although there was no clear trend in general wards, those situated in academic hospitals managed to reduce HAI rates under strict NPPI protocols. Operative surgery settings, unlike psychiatric settings, effectively prevented COVID-19 HAI using tailored NPPIs.

**Conclusion:**

The available evidence shows a high risk of COVID-19 HAIs, the feasibility of preventing HAIs in different healthcare settings and the importance of appropriately tailored NPPIs. There were no data from low-income settings, therefore, it is unclear whether the reported NPPI approaches could be equally effective elsewhere. We recommend routine monitoring of COVID-19 HAIs in countries with low vaccination coverage, to identify and close gaps in NPPIs and understand gains made from vaccinating healthcare workers and hospitalized patients.

**Supplementary Information:**

The online version contains supplementary material available at 10.1186/s12879-022-07128-5.

## Background

Coronavirus disease 2019 (COVID-19), caused by Severe Acute Respiratory Syndrome Coronavirus 2 (SARS-CoV-2) has been reported in 250 million people and caused over 5 million deaths worldwide since its outbreak in December 2019 (https://covid19.who.int/). This has led to ground-breaking turnaround times for vaccine development which saw the first batch of vaccines being rolled out in less than 15 months, by building on scientific lessons from previous SARS outbreaks [1]. Nearly three billion persons have been fully vaccinated worldwide (https://covid19.who.int/). Despite the fact that most of the currently used vaccines have been able to reduce severe disease and fatality rates, some challenges still remain, including: (i) waning immunity and less than 100% effective protection from infection, re-infection and transmission, thus requiring booster doses [2–4], (ii) the emergence of new variants, some of which are less sensitive to the current vaccine immunogens [5–7], (iii) vaccine hesitancy which in some cases is motivated by vaccine side effects [8–10], (iv) vaccine manufacturing burden which is failing to meet the population demand timeously [9] and (v) country-level financing to procure enough vaccines [11]. Low-middle-income countries (LMICs), particularly in Africa, have lagged behind in the vaccinations against SARS-CoV-2 infections (https://ourworldindata.org/covid-vaccinations) [11].

The implementation of non-pharmaceutical prevention interventions (NPPIs) becomes a critical priority especially in LMICs where vaccination coverage is extremely low. Public healthcare facilities for example are hotspots of rapid spread of COVID-19 disease if appropriate prevention protocols are not implemented and adhered to diligently [12, 13]. Both person-person and contaminated environmental surfaces-to-person spread have been reported in hospital settings [14–17]. While many LMICs include healthcare workers among their priority groups for vaccination, the congestion in many hospitals combined with inadequate infrastructural and human resources are a cause for concern in terms of patient flow and hospitalizations for COVID-19 and non-COVID-19 related illnesses. Despite the obvious slow vaccination coverage in LIMCs particularly those in Africa, there are no systems to properly monitor performance of healthcare facilities in preventing COVID-19 spread amongst healthcare workers and hospitalized patients. A hospital surveillance of COVID-19 hospital acquired infections (HAIs) in the United Kingdom showed potentially huge benefits in regularly conducting such exercises, including the ability to mitigate in-hospital transmission chains, earmarking healthcare settings at high risk of COVID-19 super-spreading and hence promoting regular review of tailored NPPIs [13]. This becomes very urgent to complement vaccination efforts, given the persisting circulation of SARS-CoV-2 worldwide.

Monitoring COVID-19 spread within healthcare centers needs to be prioritized not only to ensure effective NPPIs but also to understand the ecological benefits of COVID-19 vaccination campaigns over time, and any gaps thereof. Understanding the burden of HAIs and how they are introduced and spread in a healthcare setting will arguably promote increased vaccination coverage amongst healthcare workers, who in turn by virtue of their role, can influence vaccine acceptance amongst patients and ultimately minimize disease burden [10]. COVID-19 HAIs amongst healthcare workers in the early stage of the pandemic were widespread and reported mostly in high income countries [17]. However, the risk of COVID-19 HAI amongst patients is not as clear. We aimed to conduct a scoping review of the current evidence of COVID-19 HAIs amongst patients, with a special interest in LMICs before vaccination rollout began at the end of 2020.

## Methods

This scoping review was conducted in accordance with the PRISMA extension for Scoping Reviews guidelines (Additional file 1) [18].

### Eligibility criteria

Eligibility criteria for studies was formulated using the Population-Exposure-Comparison-Outcome-Time (PECOT) [19]. We included peer-reviewed primary studies of experimental and descriptive designs. The inclusion criteria were as follows: Participants—patients receiving health care of any kind; Exposure—admission at a healthcare facility of any type and in any country; Comparison—not important for this study; Outcome —COVID-19 positive result using any approved test; Time—before the availability of COVID-19 vaccines. The exclusion criteria were: studies reporting HAIs in healthcare workers only, non-English publications, non-peer reviewed grey literature, systematic and scoping reviews. Given the varied range of descriptive studies conducted during the rapid response to the COVID-19 pandemic, the most applicable definition for HAI with respect to the pathogenesis of COVID-19 was not applied in the eligibility criteria. One example of a strict definition for any hospital-acquired (i.e., nosocomial) infection of a patient is: ‘a positive test/symptoms within 48 h of hospitalization or within 3 days of discharge or within 30 days after an operation’ [20]. A strict definition which has been used for COVID-19 nosocomial infection of patients based on the pathogenesis profile of SARS-CoV-2 is: ‘a positive SARS-CoV-2 Reverse transcriptase polymerase chain reaction (RT-PCR) result on hospital day 3 or later or within 14 days of discharge’ [21]. It was not expected for many studies to adhere to this definition given many challenges and delays in acquiring and maintaining sufficient RT-PCR diagnostic resources in some countries, for example. The inclusion criterion for the exposure was therefore relaxed in this scoping review, in cognisant of these obvious limitations, rather, the definitions and criteria used by authors to refer to ‘COVID-19 HAI’ were accepted.

### Search strategy

An initial search of PubMed, Scopus, Web of Science, Cochrane library and Google scholar for grey literature was conducted to assess the availability of information and the types of terminology used to describe the Outcome, Participants and Exposure of interest. This output was used to formulate the search strategy using identified key words combined using Boolean operators. The initial search strategy was piloted for each database in duplicate by two authors during May 2021, and was supervised by information management expert author NL through discussions with the author pairs running the search, practical review of the search results, and reiterations of search strategies, until consistent results were obtained and agreed upon by senior authors NKN, NL and DN. On June 1, 2021, one author used the finalized search strategies and conducted the final search in PubMed, Scopus and Web of science. The search terms for Participant included: ‘patients’ OR ‘hospitalized’; Exposure search terms included: ‘hospital acquired’ OR ‘nosocomial’ OR ‘healthcare associated’. Outcome search terms included queries that mention COVID-19 and SARS-CoV-2 related key words: ‘Wuhan coronavirus’ OR ‘2019-nCoV’ OR ‘coronavirus disease 2019’ OR ‘severe acute respiratory syndrome coronavirus 2’. The comprehensive list of key words and search terms is provided in Additional file 2.

### Study selection

The search output was imported into RAYYAN software for screening [22]. Duplicate records were removed within RAYYAN prior to screening. The screening of titles and abstracts was conducted from 3 June 2021 to 20 August 2021. Two pairs of authors (TMM & RD; AT & DN) supervised by NKN, independently screened each record (title and abstract) to identify potential eligible articles using the Participant, Exposure and Outcome criteria. The title/abstract was classified as either ‘Include’, ‘exclude’ or ‘maybe’ within the RAYYAN tool to shortlist articles for full text review. Three separate databases corresponding to abstracts classified by both reviewers as ‘include’ or ‘maybe’ or had conflicting classifications, i.e., ‘conflict’, were exported into EndNote. Abstracts classified as ‘maybe’ and with disagreement between the screening pair were discussed and resolved with the lead author (NKN). Full texts for the included records were retrieved and the same peer-screening and supervision process to assess the eligibility of titles and abstracts were followed to screen the full texts.

### Data extraction

The author team developed a data extraction form, adapted from the EPOC Good Practice Data Collection guidelines (EPOC 2017b), and piloted it with five randomly selected shortlisted articles [23]. The data extraction items included author and publication year, date and country of data collection, type of healthcare facility and setting (i.e., clinical service/illnesses), age distribution of study sample population, definition used for the exposure, measure and type of the outcome, any factors associated with the outcome and recommended prevention strategies. The final tool was used independently by two reviewers (TMM & NKN) to extract the data, between 16 September and 20 October 2021. Disagreements were discussed and resolved between the reviewers and further reviewed and supervised by a third author DN.

### Data synthesis and analysis

We summarised the extracted data descriptively in Microsoft Excel. We presented ranges of estimates of COVID-19 HAIs by timing of data collection relative to implementation of prevention protocols and by the type of healthcare setting. In addition, the data were collated by country and sampled age-groups. We further discussed the external validity of observed results and the definitions used for ‘hospital-acquired’ infections with respect to SARS-CoV-2.

### Quality assessment

Use of the JBI checklist for quality of evidence was explored [24]. The study design of each eligible article was critically reviewed against the corresponding JBI checklist to obtain a quality score.

## Results

**Fig. 1:**
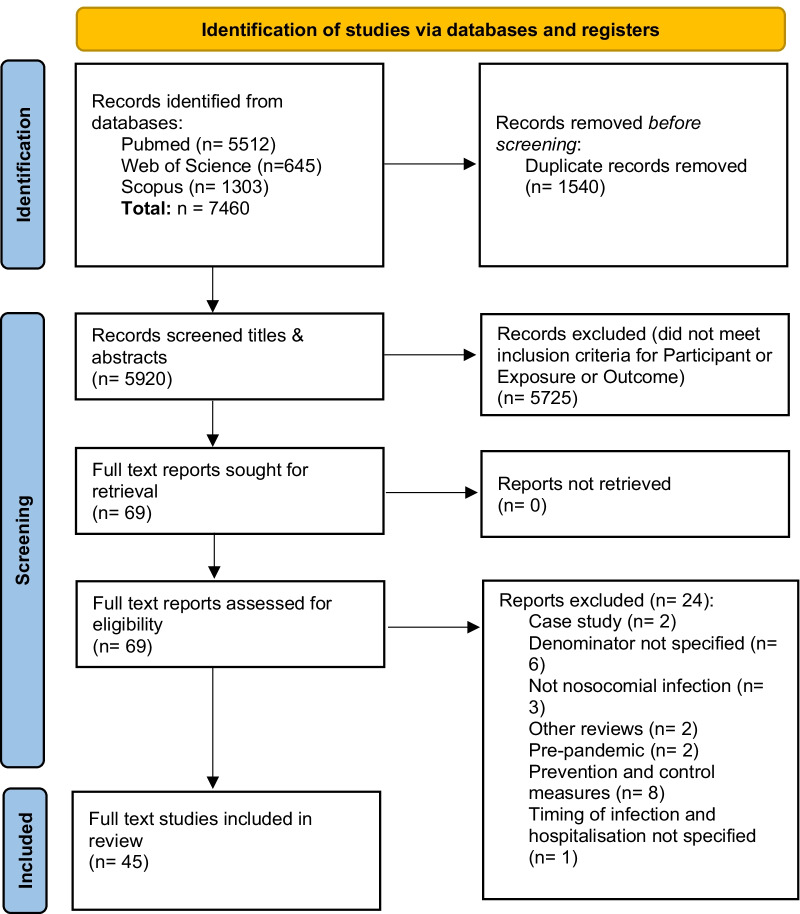
PRISMA Summary of data screening and selection process

The search strategy conducted in PubMed, Web of Science and Scopus yielded a total of 7460 potential publications. A total of 5920 titles and abstracts were screened after removing 1540 duplicates. The PRISMA flow diagram is presented in Fig. 1 and the outputs of the search strategies in Additional file 2 [25, 26]. Out of the 5920 titles and abstracts screened, 69 met the Participant, Exposure and Outcome inclusion criteria. Full texts of the 69 potentially eligible studies were downloaded and reviewed, and of these, 45 studies were included for analysis. One study reported data collected in December 2020 and 44 studies reported data collected between January–September 2020.

### Quality of evidence

The use of JBI checklist for quality of evidence could not be used for any of the included studies due to the nature of convenience sampling and study designs tailored for outbreak investigation and to provide urgent response to control the spread of COVID-19 [24]. For example, although many studies were described by authors as cohort designs, they did not meet the epidemiology definition of a cohort. However, the studies were observational in nature using some form of retrospective or prospective cohort approaches, outbreak investigation descriptions or cross-sectional reports. None of the studies performed a sample size calculation to report on both internal and external validity of their results. Some sample sizes were as small as 11 yet some were large enough to offer some level of external validity such as hospital sample sizes of over 6000 patient records.

### Available evidence of COVID-19 HAI

The included studies (Additional file 3) were conducted in 17 countries in Europe, Asia and North America, and including one study from Brazil. The highest number of studies at country-level was from the United Kingdom (UK) (10/45) followed by the United States of America (USA) (6/45). Besides Brazil, no studies from South America, Australia, and Africa, met the inclusion criteria for this review. Most of the data were collected between March and May 2020 from a diverse array of healthcare settings including long-term care facilities, dialysis units, cancer and other operative surgery wards including neurology units, psychiatric centers, academic and general hospitals. Age groups varied, although most studies reported data from adults over the age of 50 years.

COVID-19 HAIs were largely reported as simple proportions out of the total number of screened hospitalized patients. Only two studies reported incidence rates: a study in France reported a COVID-19 HAI rate of 155.6 cases per 100,000 in-hospitalized patients [27], and another in the USA [28] reported an HAI rate of 0.8–5.0 cases per 10,000 patient days. The remaining studies reported proportions between 0 and 65.00%. Studies with similar sample age-groups did not necessarily report similar estimates of HAIs either (Additional file 4). The 29 studies with adults-only samples reported a wide range of HAI estimates from 0 to 65.00%. The estimates from the two studies reporting on children/neonates also fell within this range, i.e., 27.08–35.00%.

### HAI estimates by country and pandemic phases

Four studies did not provide specific dates for the data collection period. Out of the remaining 41 studies, 95% (39/41) initiated data collection before May 2020 during the very early stage of the global pandemic and 37 of them completed data collection by June 2020 (Additional file 3). Besides the remaining 2/41 studies conducted in July (Middle East) and December 2020 (China), there were no studies identified after this early phase. It was difficult to compare estimates between countries due to the varying number of studies but appeared to overlap between countries in cases where several studies were reported. For example, the ranges of HAI estimates were 6.48–35.00%, 0–46.29% and 0.02–53.60% in Spain, UK, and USA, respectively (Additional file 5). Within the North American region, Canada had a lower range of estimates (4.60–19.00%) but from only two studies compared to the USA (0.02–53.60%) with six studies. Other European countries, besides the UK and Spain, reported results from only one, two or three studies and the estimates were anything between 0.16 and 65.00%. Although most studies were conducted over the same time-period and overlapping calendar months, the HAI estimates were not similar between studies from the same country, e.g., the two studies in Belgium reported HAI proportions of 2.84% (conducted in March–May 2020) and 65.00% (conducted in March–April 2020).

### HAI estimates by timing of implementing prevention measures and type of hospital service

COVID-19 HAI estimates appeared to differ by timing of implementation of NPPIs relative to when a study was conducted (Table 1) as well as by type of healthcare service (Table 2). Only 3/14 studies conducted before the implementation of NPPIs at study sites, reported low (< 7%) HAI proportions while the remainder 11/14 studies reported proportions between 18.50 and 65.00% (Table 1). On the contrary, the majority (18/22) of studies conducted post-initiation of NPPIs at study sites, reported low (< 7%) HAIs proportions and only 4/22 reported high proportions between 15.40 and 53.60% (Table 1).

**Table 1:** Estimates of COVID-19 hospital acquired infections grouped by timing of prevention protocols

Author, publication date	Total sample size of patients included in analysis	Proportion of HAI (or incidence rate)	Study timing relative to NPPIs
Ajayi 2020	657	4.10%	Before
Luong-Nguyen 2020	301	4.90%	Before
Lakhani 2020	288	6.48%	Before
Harada 2020	562	18.50%	Before
Borges 2021	92	22.83%	Before
Lio 2021	28	25.00%	Before
Schwierzeck 2020	48	27.08%	Before
Bestillieiro 2020	34	29.41%	Before
Romero 2020	198	33.60%	Before
Constantino-Shor 2021	25	40.00%	Before
Davis 2021	222	46.29%	Before
Horing 2020	50	52.00%	Before
Caronni 2021	11	54.50%	Before
Mazzoleni 2020	62	65.00%	Before
Romics 2021	179	0.00%	During
Rhee 2020	9149	0.02%	During
Ambrosch 2020	6106	0.16%	During
Sahoo 2020	1769	0.28%	During
Mettias 2020	3410	0.32%	During
Demiroz 2020	162	1.20%	During
Romao 2020	617	1.30%	During
Tabourin 2020	68	1.50%	During
Axiotakis 2021	501	1.80%	During
Wee 2020	45	2.20%	During
Lubansu 2020	176	2.84%	During
Jeannon 2021	69	3.00%	During
Rajasekaran 2021	347	4.00%	During
Yau 2020	237	4.60%	During
Meena 2020	20	5.00%	During
Sobrado 2020	99	5.00%	During
Jewkes 2020	133	6.00%	During
Khonyongwa 2020	774	7.10%	During
Cheng 2021	78	15.40%	During
Thompson 2020	160	33.00%	During
Al Lawati 2020	28	46.43%	During
Goldberg 2020	97	53.60%	During
Long 2021	2992	0.8–5.0 cases per 10,000 patient days	During
Biernat 2020	39	4.87%	Not clear
Khan 2021	173	11.00%	Not clear
Carter 2020	1564	12.50%	Not clear
Bhogal 2020	179	16.00%	Not clear
Garratti 2020	52	19.00%	Not clear
Elkrief 2020	252	19.00%	Not clear
Colomer 2020	40	35.00%	Not clear
Gaudart 2021	100,988	155.6 cases (range 19·4–489.5) per 100,000 in-hospital habitants	Not clear

Lists of studies conducted before, during or at unknown timing in relation to the implementation of non-pharmaceutical prevention interventions (NPPIs) at study sites. Studies are listed in order of increasing HAI proportions within each group. HAI, hospital acquired infections

**Table 2:** Estimates of COVID-19 hospital acquired infections grouped by type of healthcare service

Hospital service	Author	Proportion of HAI, %	HAI incidence rate
Cancer	Romics 2021	0	
	Jeannon 2021	3.00	
	Rajasekaran 2021	4.00	
	Biernat 2020	4.87	
	Luong-Nguyen 2020	4.90	
	Sobrado 2020	5.00	
	Bhogal 2020	16.00	
	Elkrief 2020	19.00	
	Bestillieiro 2020	29.41	
Dialysis	Yau 2020	4.60	
	Lio 2021	25.00	
	Schwierzeck 2020	27.08	
	Mazzoleni 2020	65.00	
Long-term care	Romero 2020	33.60	
	Goldberg 2020	53.60	
Neurological	Sahoo 2020	0.28	
	Lubansu 2020	2.84	
	Jewkes 2020	6.00	
	Caronni 2021	54.50	
Paediatrics	Colomer 2020	35.00	
Psychiatry	Thompson 2020	33.00	
	Constantino-Shor 2021	40.00	
Operative surgery	Mettias 2020	0.32	
	Demiroz 2020	1.20	
	Axiotakis 2021	1.80	
	Lakhani 2020	6.48	
	Garratti 2020	19.00	
Rheumatoid Illness	Romao 2020	1.30	
General wards/other	Gaudart 2021		155.6 cases per 100,000 in-hospital habitants
	Long 2021		0.8–5.0 cases per 10,000 patient days
	Rhee 2020	0.02	
	Ambrosch 2020	0.16	
	Tabourin 2020	1.50	
	Wee 2020	2.20	
	Ajayi 2020	4.10	
	Meena 2020	5.00	
	Khonyongwa 2020	7.10	
	Khan 2021	11.00	
	Carter 2020	12.50	
	Cheng 2021	15.40	
	Harada 2020	18.50	
	Borges 2021	22.83	
	Davis 2021	46.29	
	Horing 2020	52.00	
	Al Lawati 2020	56.43	

Lists of studies conducted in different healthcare services. Studies are listed in order of increasing HAI proportions within each group. HAI, hospital acquired infections

Low estimates (< 7%) of COVID-19 HAIs were also observed in most of the studies conducted in healthcare settings servicing cancer patients (6/9), neurology (3/4) and other operative surgery (4/5) (Table 2). Two of these studies (one in a cancer setting and the other in operative surgery) were amongst the only three with low HAIs but conducted before NPPIs were implemented at study sites [29–31]. Although there were fewer studies grouped by other healthcare services, most studies in psychiatry hospitals (2/2), dialysis units (3/4) and long-term care (2/2), reported high HAI proportions, all over 25.00% (Table 2). Two of these studies, one conducted in psychiatry [24], and the other in a long-term care setting [25], were amongst the only four studies with high HAIs after initiation of NPPIs at study sites. The remainder two studies reporting high HAIs during use of NPPIs were conducted in general hospital wards. One of these was conducted twelve months into the pandemic in China and this unexpected outcome was related to a possible relaxation in adherence to protocols amongst hospital personnel [32].

## Discussion

In this scoping review on COVID-19 HAIs amongst hospitalized patients, no data was found from LMICs where most of the countries have the lowest coverage of COVID-19 vaccinations and hence the highest risk of continued spread of SARS-CoV-2 in enclosed close-contact spaces such as hospitals. The data, mostly from high-income settings, show that COVID-19 HAI did not differ by country, instead there appeared to be a pattern related to the timing of prevention interventions and the type of healthcare service. Overall, although it was possible to completely avoid COVID-19 HAIs, the data implied that without strict adherence to NPPIs, COVID-19 HAI infections can easily spread to catastrophically high levels, in this case reaching two thirds of hospitalized patients sharing a ward. This observed level of risk of spread of COVID-19 HAIs is very high compared to that of HAIs of other respiratory infections. For example, a systematic review of hospital acquired influenza reported proportions ranging between 6 and 13% [33]. High income countries specifically, have reported nosocomial infections of other respiratory viral infections of around 3.9 cases or lower per 1 000 hospitalized patients, for instance [34, 35]. In LMIC settings, nosocomial infections of other respiratory infections have been shown to reach 13.5% [36].

Evidence from this review shows that the implementation of NPPIs in high-income settings is effective in reducing the spread of COVID-19 HAIs, and in some cases to completely avoid it. The nature of healthcare service appears to influence the ease or complexity with which tailored NPPIs can be introduced and adhered to. Operative surgeries including specific examples like neurology and cancer treatment units, appeared to perform well in terms of controlling COVID-19 HAIs, probably due to their routines of high level hygienic practises with or without COVID-19 [29–31]. In other hospital settings, such as haemodialysis, the risk of COVID-19 HAI spread was high but adherence to strict prevention protocols appeared to be feasible and very effective in reducing HAIs [37–39]. General care wards in well-resourced academic hospitals also appeared to achieve effective control measures and significant reductions in HAIs [40–42]. However, in some cases it may not be as straightforward, such as in a psychiatric hospital attempting to follow CDC guidelines, where infection rates decreased but the levels remained of public health concern and as high as in a similar setting prior to implementing NPPIs [43]. These observations indicate, in addition to adopting universal NPPIs, that protocols tailored to the type of healthcare service setting are needed. Unfortunately, there is no data to understand similar healthcare scenarios in low-resourced hospital settings. All the results included in this study are from the pre-vaccination period (February–September 2020) of the COVID-19 pandemic and could still be relevant in countries where vaccination coverage is low, as well as important to use as reference points for post-vaccination surveillance.

### Recommendations for preventing super-spreading COVID-19 in healthcare centers

Some recommendations for tailored NPPIs to maximize efforts to prevent HAIs were noted from the reviewed studies and could be adopted across the globe. These include the following: In all healthcare settings, adherence to strict spatial separation of patient beds and frequent intensified training of healthcare workers are recommended alongside the standard recommended prevention procedures [44]. Cleaning of touch surfaces was suggested to be as frequent as every two hours in order to be effective [45]. Strategies to reduce airborne transmission included use of portable particulate filters in wards and regular meticulous cleaning of non-invasive ventilation apparatus which have been reported to spark super-spreading events [32, 46, 47]. Universal testing of COVID-19 infection on admission appeared to be effective in lowering HAIs, with some hospitals recommending daily screening and testing of symptomatic hospital habitants [41, 45]. This requires properly designed holding wards while waiting for results, without promoting spread amongst these waiting patients. Whether this is feasible in low-resource and high-volume hospital settings is of concern.

In operative surgeries, universal recommendations for different types of surgery operations included screening and testing within 48–72 h pre-operative; isolation and delayed elective/non-urgent procedures if symptomatic, and only proceeding after a confirmed negative RT-PCR test result [28, 48, 49]. Given the short period of monitoring before surgery, two different sources of swabs (e.g., throat and nasal) or a swab and a chest X-ray appeared to be preferred for accuracy of COVID-19 diagnosis [50]. Delay in surgery procedures by at least 14 days if symptomatic or conducting double sequential testing for those who are asymptomatic was also recommended [51, 52]. Post-operative monitoring with at least one additional RT-PCR test is recommended for at least 14 days and until 30 days post-operative [28, 48, 49]. The recommended measures were reported to be effective and observed perioperative HAIs in this context were associated with specific risk factors including complicated procedures such as transplant surgery or presence of chronic comorbidities, advanced malignancies and recent chemotherapy [49, 53].

If periodic monitoring of adherence to prevention measures, review and re-strengthening are not performed, there is a chance of re-introduction of outbreaks [54]. The issue regarding asymptomatic healthcare workers and patients with delayed viral shedding, which could lead to unexpected outbreaks even in well-controlled healthcare centers, remains a concern [55, 56]. Vaccinating everyone could be the best and only solution for these concerns, further emphasizing the critical urgency of increasing vaccine access and coverage across LMICs, where effectively maintaining NPPIs may not be that easy in many hospital settings. The waning of vaccine-induced immunity against COVID-19 also emphasizes the need for monitoring and evaluation of NPPIs in healthcare settings to ensure these are tailored appropriately and adhered to. Monitoring the rate of COVID-19 HAI as part of routine practice, could be another approach to evaluate the effectiveness of NPPIs as well as the gains made from the vaccination campaigns of healthcare workers and hospitalized patients.

### Limitations

Grey literature was not included in the study. Only peer reviewed published manuscripts written in English were considered. However, peer reviewed manuscripts provide more reliable scientific evidence and are the sources referenced by policy guidelines.

Other limitations observed are not from the study design and methodology but from the observed retrieved literature. The first is the fewer number of studies from most countries, making it impossible to understand inter-country differences, if there were any. The second is the inability to understand the possible influence of different pandemic phases because nearly all studies were conducted during the same and earliest period of the pandemic. Thirdly, the studies fell short in terms of epidemiologically rigorous research designs and reporting measures, hence quality assessment to report the strength of evidence could not be conducted.

### Generalizability

None of the included studies conducted sample size and power calculations, probably due to the rapid outbreak response conditions. Although the observed results are useful to understand the importance of adhering to strict prevention protocols in healthcare centers, there is not direct external validity to inform specific scenarios of low-income settings.

Most of the results with clear definitions of nosocomial infections and meticulous prospective surveillance systems were from well-resourced academic hospitals. The heterogeneity observed in the definitions used for HAI can also make it difficult to compare estimates from different settings. HAI definitions used for many operative surgeries were consistent and relevant to the nature of healthcare service provided, for example, a negative result 48–72 h pre-operative followed with a positive result during hospital stay 2–14 days post-operative[48–50]. Definitions varied widely in other healthcare service settings. Although some studies defined HAIs based on the median incubation period of SARS-CoV-2 known at the time of the outbreak, a negative diagnosis result at point of admission was not confirmed for example [27, 57]. However, other studies confirmed negative infection status at point of hospital admission followed with a positive result within 14 or more days of hospitalization and until 14 days after discharge [30, 58]. Given current knowledge about the pathogenesis profile of SARS-CoV-2 in humans, we recommend that the definition of COVID-19 HAI should include confirmed uninfected status during the first 72 h of hospital admission, followed by a positive result thereafter during hospitalization or until 14–30 days post-discharge [20, 45, 59]. In addition, given the wide range of the possible incubation period, we recommend including the approach used by Bhogal et al., 2020, to further stratify HAI into ‘definite’, ‘probable’ or ‘indeterminant’ for > 14 days, 8–14 days and 3–7 days after admission, respectively [53]. HAIs diagnosed post-discharge could be described as 0–7 days, 8–14 days, 15–30 days post-discharge for ‘definite’, ‘probable’ and ‘indeterminant’, respectively.

### The importance of vaccination

Evidence is clear that vaccination reduces the risk of severe COVID-19 disease and mortality and it could, speculatively, also reduce person-to-environmental spread of high viral load [2, 60–62]. While NPPIs are essential for as long as the COVID-19 pandemic persists, vaccination as known historically [63], is the key to curbing the spread of SARS-CoV-2. General care hospitals could be the ideal points to monitor the need for and effectiveness of booster doses due to the high turnaround of patients, combined with the daily in and out shifts of healthcare staff [62, 64, 65]. The hospital setting also provides an ideal place to assess the benefits of high vaccination coverage in minimising person-environmental surface preservation of viruses. Periodic random sampling of different types of healthcare settings could be useful to monitor the ecological effectiveness of increasing vaccination coverage and of increasing booster dose coverage.

## Conclusions

The risk of COVID-19 HAIs is very high, but infections can be prevented provided mitigation protocols are customized to the type of hospital service and setting and regular monitoring and evaluation of adherence and effectiveness is conducted. None of the identified studies were from countries in low-income settings, therefore the feasibility of prevention practices in low-resourced settings is unclear. There is work needed to evaluate the performance of public healthcare hospitals in preventing COVID-19 HAIs in low-income settings, particularly in countries where the coverage of vaccination against COVID-19 is still low. We recommend: (i) conduct of high quality observational study designs embedded in routine healthcare settings to provide reliable evidence on HAIs in LMICs, (ii) COVID-19 vaccination hubs or promotion points to be available at every healthcare center in LMICs to increase expedited vaccination coverage amongst all staff, visitors, outpatients, short-term and long-term care patients and (iii) countries still with low vaccination coverage monitor COVID-19 HAIs as part of routine practice in healthcare centers, to strengthen NPPIs and understand gains being made from increasing COVID-19 vaccination coverage.

## Supplementary Information

**Additional file 1: Table S1.** PRISMA checklist for scoping reviews.

**Additional file 2: Table S2.** Search strategies.

**Additional file 3: Table S3.** Summary of studies included in the scoping review.

**Additional file 4: Table S4.** Estimates of COVID-19 hospital acquired infections grouped by patient age-groups.

**Additional file 5: Table S5.** Estimates of COVID-19 hospital acquired infections grouped by country.

## Data Availability

All data generated or analysed during this study are included in this published article [and its Additional files].

## Abbreviations

COVID-19: Coronavirus Disease 2019
HAIs: Hospital acquired infections
LMICs: Low-middle-income countries
NPPIs: Non-pharmaceutical prevention interventions
RT-PCR: Reverse transcriptase polymerase chain reaction
SARS-CoV-2: Severe Acute Respiratory Syndrome Coronavirus 2
