# Supplementary material for: Hospital acquired COVID-19 infections amongst patients before the rollout of COVID-19 vaccinations, a scoping review
Source: BMC Infect Dis. 2022 Feb 10;22:140. doi: 10.1186/s12879-022-07128-5 (PMC8830001; doi:10.1186/s12879-022-07128-5)
Supplement: Supplementary file 2 — Additional file 2: Table S2. Search strategies. [file 12879_2022_7128_MOESM2_ESM.docx]

| **PUBMED SEARCH STRATEGY 1 JUNE 2021** | | |
| --- | --- | --- |
| **Search** | **Query** | **Results** |
| #8 (#7 filtered to 1 Oct 2019 to 1 June 2021) | Search: ((coronavirus[mh:noexp] OR coronavirus*[tiab] OR corona virus*[tiab] OR COVID-19[mh] OR covid-19[tiab] OR covid19[tiab] OR covid 2019[tiab] OR SARS-Cov-2[mh] OR SARS-CoV-2[tiab] OR SARS-CoV2[tiab] OR SARSCoV2[tiab] OR SARsCov-2[tiab] OR SARS-coronavirus*[tiab] OR severe acute respiratory syndrome coronavirus 2[nm] OR severe acute respiratory syndrome coronavirus 2[tiab] OR 2019-nCov[tiab] OR 2019nCov[tiab] OR nCov2019[tiab] OR nCOV-2019[tiab] OR hCOV*[tiab] OR n-cov[tiab] OR ncov*[tiab] OR coronaviridae*[tiab] OR betacoronavirus*[tiab] OR betacoronaviruses*[tiab] OR sars cov 2[tiab] OR novel CoV[tiab] OR wuhan virus*[tiab] OR hubei[tiab] OR SARS2[tiab] OR corona virus*[tiab] OR sars*[tiab] AND (2019:2021[pdat])) AND (Cross infection[mh] OR cross infection*[tiab] OR hospital infection*[tiab] OR nosocomial*[tiab] OR health care associated infection*[tiab] OR healthcare associated infection*[tiab] OR healthcare infection*[tiab] OR healthcare acquired[tiab] OR hospital acquired[tiab] OR hospital associated[tiab] OR hospital incidence[tiab] OR healthcare incidence[tiab] OR health care incidence[tiab] OR hospital case*[tiab] OR hospital spread*[tiab] OR healthcare case*[tiab] OR healthcare spread*[tiab] AND (2019:2021[pdat]))) AND (Patient*[tiab] OR hospitalised*[tiab] OR hospitalized*[tiab] OR admitted*[tiab] AND (2019:2022[pdat])) Filters: from 2019/10/1 - 2021/6/1 | [5,512](https://pubmed.ncbi.nlm.nih.gov/?term=longquerya8fff0db66e662588cde&filter=dates.2019%2F10%2F1-2021%2F6%2F1&size=200&sort=relevance) |
| #7 (#2 AND #4 AND #6) | Search: ((coronavirus[mh:noexp] OR coronavirus*[tiab] OR corona virus*[tiab] OR COVID-19[mh] OR covid-19[tiab] OR covid19[tiab] OR covid 2019[tiab] OR SARS-Cov-2[mh] OR SARS-CoV-2[tiab] OR SARS-CoV2[tiab] OR SARSCoV2[tiab] OR SARsCov-2[tiab] OR SARS-coronavirus*[tiab] OR severe acute respiratory syndrome coronavirus 2[nm] OR severe acute respiratory syndrome coronavirus 2[tiab] OR 2019-nCov[tiab] OR 2019nCov[tiab] OR nCov2019[tiab] OR nCOV-2019[tiab] OR hCOV*[tiab] OR n-cov[tiab] OR ncov*[tiab] OR coronaviridae*[tiab] OR betacoronavirus*[tiab] OR betacoronaviruses*[tiab] OR sars cov 2[tiab] OR novel CoV[tiab] OR wuhan virus*[tiab] OR hubei[tiab] OR SARS2[tiab] OR corona virus*[tiab] OR sars*[tiab] AND (2019:2021[pdat])) AND (Cross infection[mh] OR cross infection*[tiab] OR hospital infection*[tiab] OR nosocomial*[tiab] OR health care associated infection*[tiab] OR healthcare associated infection*[tiab] OR healthcare infection*[tiab] OR healthcare acquired[tiab] OR hospital acquired[tiab] OR hospital associated[tiab] OR hospital incidence[tiab] OR healthcare incidence[tiab] OR health care incidence[tiab] OR hospital case*[tiab] OR hospital spread*[tiab] OR healthcare case*[tiab] OR healthcare spread*[tiab] AND (2019:2021[pdat]))) AND (Patient*[tiab] OR hospitalised*[tiab] OR hospitalized*[tiab] OR admitted*[tiab] AND (2019:2022[pdat])) | [5,533](https://pubmed.ncbi.nlm.nih.gov/?term=%28%28coronavirus%5Bmh%3Anoexp%5D+OR+coronavirus%2A%5Btiab%5D+OR+corona+virus%2A%5Btiab%5D+OR+COVID-19%5Bmh%5D+OR+covid-19%5Btiab%5D+OR+covid19%5Btiab%5D+OR+covid+2019%5Btiab%5D+OR+SARS-Cov-2%5Bmh%5D+OR+SARS-CoV-2%5Btiab%5D+OR+SARS-CoV2%5Btiab%5D+OR+SARSCoV2%5Btiab%5D+OR+SARsCov-2%5Btiab%5D+OR+SARS-coronavirus%2A%5Btiab%5D+OR+severe+acute+respiratory+syndrome+coronavirus+2%5Bnm%5D+OR+severe+acute+respiratory+syndrome+coronavirus+2%5Btiab%5D+OR+2019-nCov%5Btiab%5D+OR+2019nCov%5Btiab%5D+OR+nCov2019%5Btiab%5D+OR+nCOV-2019%5Btiab%5D+OR+hCOV%2A%5Btiab%5D+OR+n-cov%5Btiab%5D+OR+ncov%2A%5Btiab%5D+OR+coronaviridae%2A%5Btiab%5D+OR+betacoronavirus%2A%5Btiab%5D+OR+betacoronaviruses%2A%5Btiab%5D+OR+sars+cov+2%5Btiab%5D+OR+novel+CoV%5Btiab%5D+OR+wuhan+virus%2A%5Btiab%5D+OR+hubei%5Btiab%5D+OR+SARS2%5Btiab%5D+OR+corona+virus%2A%5Btiab%5D+OR+sars%2A%5Btiab%5D+AND+%282019%3A2021%5Bpdat%5D%29%29+AND+%28Cross+infection%5Bmh%5D+OR+cross+infection%2A%5Btiab%5D+OR+hospital+infection%2A%5Btiab%5D+OR+nosocomial%2A%5Btiab%5D+OR+health+care+associated+infection%2A%5Btiab%5D+OR+healthcare+associated+infection%2A%5Btiab%5D+OR+healthcare+infection%2A%5Btiab%5D+OR+healthcare+acquired%5Btiab%5D+OR+hospital+acquired%5Btiab%5D+OR+hospital+associated%5Btiab%5D+OR+hospital+incidence%5Btiab%5D+OR+healthcare+incidence%5Btiab%5D+OR+health+care+incidence%5Btiab%5D+OR+hospital+case%2A%5Btiab%5D+OR+hospital+spread%2A%5Btiab%5D+OR+healthcare+case%2A%5Btiab%5D+OR+healthcare+spread%2A%5Btiab%5D+AND+%282019%3A2021%5Bpdat%5D%29%29%29+AND+%28Patient%2A%5Btiab%5D+OR+hospitalised%2A%5Btiab%5D+OR+hospitalized%2A%5Btiab%5D+OR+admitted%2A%5Btiab%5D+AND+%282019%3A2022%5Bpdat%5D%29%29&sort=&size=200) |
| #6 (Date filter: 2019-2021) | Search: Patient*[tiab] OR hospitalised*[tiab] OR hospitalized*[tiab] OR admitted*[tiab] Filters: from 2019 - 2022 | [1,071,682](https://pubmed.ncbi.nlm.nih.gov/?term=Patient%2A%5Btiab%5D+OR+hospitalised%2A%5Btiab%5D+OR+hospitalized%2A%5Btiab%5D+OR+admitted%2A%5Btiab%5D&filter=years.2019-2022&size=200&sort=relevance) |
| #5 | Search: Patient*[tiab] OR hospitalised*[tiab] OR hospitalized*[tiab] OR admitted*[tiab] | [7,184,350](https://pubmed.ncbi.nlm.nih.gov/?term=Patient%2A%5Btiab%5D+OR+hospitalised%2A%5Btiab%5D+OR+hospitalized%2A%5Btiab%5D+OR+admitted%2A%5Btiab%5D&size=200&sort=relevance) |
| #4 (Date filter: 2019-2021) | Search: Cross infection[mh] OR cross infection*[tiab] OR hospital infection*[tiab] OR nosocomial*[tiab] OR health care associated infection*[tiab] OR healthcare associated infection*[tiab] OR healthcare infection*[tiab] OR healthcare acquired[tiab] OR hospital acquired[tiab] OR hospital associated[tiab] OR hospital incidence[tiab] OR healthcare incidence[tiab] OR health care incidence[tiab] OR hospital case*[tiab] OR hospital spread*[tiab] OR healthcare case*[tiab] OR healthcare spread*[tiab] Filters: from 2019 - 2021 | [35,484](https://pubmed.ncbi.nlm.nih.gov/?term=Cross+infection%5Bmh%5D+OR+cross+infection%2A%5Btiab%5D+OR+hospital+infection%2A%5Btiab%5D+OR+nosocomial%2A%5Btiab%5D+OR+health+care+associated+infection%2A%5Btiab%5D+OR+healthcare+associated+infection%2A%5Btiab%5D+OR+healthcare+infection%2A%5Btiab%5D+OR+healthcare+acquired%5Btiab%5D+OR+hospital+acquired%5Btiab%5D+OR+hospital+associated%5Btiab%5D+OR+hospital+incidence%5Btiab%5D+OR+healthcare+incidence%5Btiab%5D+OR+health+care+incidence%5Btiab%5D+OR+hospital+case%2A%5Btiab%5D+OR+hospital+spread%2A%5Btiab%5D+OR+healthcare+case%2A%5Btiab%5D+OR+healthcare+spread%2A%5Btiab%5D&filter=years.2019-2021&size=200&sort=relevance) |
| #3 | Search: Cross infection[mh] OR cross infection*[tiab] OR hospital infection*[tiab] OR nosocomial*[tiab] OR health care associated infection*[tiab] OR healthcare associated infection*[tiab] OR healthcare infection*[tiab] OR healthcare acquired[tiab] OR hospital acquired[tiab] OR hospital associated[tiab] OR hospital incidence[tiab] OR healthcare incidence[tiab] OR health care incidence[tiab] OR hospital case*[tiab] OR hospital spread*[tiab] OR healthcare case*[tiab] OR healthcare spread*[tiab] | [185,663](https://pubmed.ncbi.nlm.nih.gov/?term=Cross+infection%5Bmh%5D+OR+cross+infection%2A%5Btiab%5D+OR+hospital+infection%2A%5Btiab%5D+OR+nosocomial%2A%5Btiab%5D+OR+health+care+associated+infection%2A%5Btiab%5D+OR+healthcare+associated+infection%2A%5Btiab%5D+OR+healthcare+infection%2A%5Btiab%5D+OR+healthcare+acquired%5Btiab%5D+OR+hospital+acquired%5Btiab%5D+OR+hospital+associated%5Btiab%5D+OR+hospital+incidence%5Btiab%5D+OR+healthcare+incidence%5Btiab%5D+OR+health+care+incidence%5Btiab%5D+OR+hospital+case%2A%5Btiab%5D+OR+hospital+spread%2A%5Btiab%5D+OR+healthcare+case%2A%5Btiab%5D+OR+healthcare+spread%2A%5Btiab%5D&size=200&sort=relevance) |
| #2 (Date filter: 2019-2021) | Search: coronavirus[mh:noexp] OR coronavirus*[tiab] OR corona virus*[tiab] OR COVID-19[mh] OR covid-19[tiab] OR covid19[tiab] OR covid 2019[tiab] OR SARS-Cov-2[mh] OR SARS-CoV-2[tiab] OR SARS-CoV2[tiab] OR SARSCoV2[tiab] OR SARsCov-2[tiab] OR SARS-coronavirus*[tiab] OR severe acute respiratory syndrome coronavirus 2[nm] OR severe acute respiratory syndrome coronavirus 2[tiab] OR 2019-nCov[tiab] OR 2019nCov[tiab] OR nCov2019[tiab] OR nCOV-2019[tiab] OR hCOV*[tiab] OR n-cov[tiab] OR ncov*[tiab] OR coronaviridae*[tiab] OR betacoronavirus*[tiab] OR betacoronaviruses*[tiab] OR sars cov 2[tiab] OR novel CoV[tiab] OR wuhan virus*[tiab] OR hubei[tiab] OR SARS2[tiab] OR corona virus*[tiab] OR sars*[tiab] Filters: from 2019 - 2021 | [140,905](https://pubmed.ncbi.nlm.nih.gov/?term=coronavirus%5Bmh%3Anoexp%5D+OR+coronavirus%2A%5Btiab%5D+OR+corona+virus%2A%5Btiab%5D+OR+COVID-19%5Bmh%5D+OR+covid-19%5Btiab%5D+OR+covid19%5Btiab%5D+OR+covid+2019%5Btiab%5D+OR+SARS-Cov-2%5Bmh%5D+OR+SARS-CoV-2%5Btiab%5D+OR+SARS-CoV2%5Btiab%5D+OR+SARSCoV2%5Btiab%5D+OR+SARsCov-2%5Btiab%5D+OR+SARS-coronavirus%2A%5Btiab%5D+OR+severe+acute+respiratory+syndrome+coronavirus+2%5Bnm%5D+OR+severe+acute+respiratory+syndrome+coronavirus+2%5Btiab%5D+OR+2019-nCov%5Btiab%5D+OR+2019nCov%5Btiab%5D+OR+nCov2019%5Btiab%5D+OR+nCOV-2019%5Btiab%5D+OR+hCOV%2A%5Btiab%5D+OR+n-cov%5Btiab%5D+OR+ncov%2A%5Btiab%5D+OR+coronaviridae%2A%5Btiab%5D+OR+betacoronavirus%2A%5Btiab%5D+OR+betacoronaviruses%2A%5Btiab%5D+OR+sars+cov+2%5Btiab%5D+OR+novel+CoV%5Btiab%5D+OR+wuhan+virus%2A%5Btiab%5D+OR+hubei%5Btiab%5D+OR+SARS2%5Btiab%5D+OR+corona+virus%2A%5Btiab%5D+OR+sars%2A%5Btiab%5D&filter=years.2019-2021&size=200&sort=relevance) |
| #1 | Search: coronavirus[mh:noexp] OR coronavirus*[tiab] OR corona virus*[tiab] OR COVID-19[mh] OR covid-19[tiab] OR covid19[tiab] OR covid 2019[tiab] OR SARS-Cov-2[mh] OR SARS-CoV-2[tiab] OR SARS-CoV2[tiab] OR SARSCoV2[tiab] OR SARsCov-2[tiab] OR SARS-coronavirus*[tiab] OR severe acute respiratory syndrome coronavirus 2[nm] OR severe acute respiratory syndrome coronavirus 2[tiab] OR 2019-nCov[tiab] OR 2019nCov[tiab] OR nCov2019[tiab] OR nCOV-2019[tiab] OR hCOV*[tiab] OR n-cov[tiab] OR ncov*[tiab] OR coronaviridae*[tiab] OR betacoronavirus*[tiab] OR betacoronaviruses*[tiab] OR sars cov 2[tiab] OR novel CoV[tiab] OR wuhan virus*[tiab] OR hubei[tiab] OR SARS2[tiab] OR corona virus*[tiab] OR sars*[tiab] | [158,654](https://pubmed.ncbi.nlm.nih.gov/?term=coronavirus%5Bmh%3Anoexp%5D+OR+coronavirus%2A%5Btiab%5D+OR+corona+virus%2A%5Btiab%5D+OR+COVID-19%5Bmh%5D+OR+covid-19%5Btiab%5D+OR+covid19%5Btiab%5D+OR+covid+2019%5Btiab%5D+OR+SARS-Cov-2%5Bmh%5D+OR+SARS-CoV-2%5Btiab%5D+OR+SARS-CoV2%5Btiab%5D+OR+SARSCoV2%5Btiab%5D+OR+SARsCov-2%5Btiab%5D+OR+SARS-coronavirus%2A%5Btiab%5D+OR+severe+acute+respiratory+syndrome+coronavirus+2%5Bnm%5D+OR+severe+acute+respiratory+syndrome+coronavirus+2%5Btiab%5D+OR+2019-nCov%5Btiab%5D+OR+2019nCov%5Btiab%5D+OR+nCov2019%5Btiab%5D+OR+nCOV-2019%5Btiab%5D+OR+hCOV%2A%5Btiab%5D+OR+n-cov%5Btiab%5D+OR+ncov%2A%5Btiab%5D+OR+coronaviridae%2A%5Btiab%5D+OR+betacoronavirus%2A%5Btiab%5D+OR+betacoronaviruses%2A%5Btiab%5D+OR+sars+cov+2%5Btiab%5D+OR+novel+CoV%5Btiab%5D+OR+wuhan+virus%2A%5Btiab%5D+OR+hubei%5Btiab%5D+OR+SARS2%5Btiab%5D+OR+corona+virus%2A%5Btiab%5D+OR+sars%2A%5Btiab%5D+&sort=&size=200) |
| **WEB OF SCIENCE SEARCH STRATEGY 1 JUNE 2021** | | |
| #4 | #3  AND  #2  AND  #1  *Indexes=SCI-EXPANDED, SSCI, A&HCI, ESCI Timespan=2019-2021* | [645](http://apps.webofknowledge.com/summary.do?product=WOS&doc=1&qid=4&SID=F5blfoj97UWodAibDV3&search_mode=CombineSearches&update_back2search_link_param=yes) |
| #3 | (TS=(“nosocomial infection” OR “cross infection” OR “hospital acquired infection” OR “healthcare associated infection” OR “healthcare-associated infections” OR “healthcare-acquired infections” OR “hospital acquired infections” OR nosocomial OR “healthcare associated” OR “hospital acquired” OR “hospital-acquired” OR “healthcare-associated” OR “healthcare spread” OR “healthcare case” OR “healthcare incidence” OR “hospital case” OR “hospital incidence” OR “hospital spread”) )  *AND* LANGUAGE:  (English)  *Indexes=SCI-EXPANDED, SSCI, A&HCI, ESCI Timespan=2019-2021* | [8,580](http://apps.webofknowledge.com/summary.do?product=WOS&doc=1&qid=3&SID=F5blfoj97UWodAibDV3&search_mode=AdvancedSearch&update_back2search_link_param=yes) |
| #2 | (TS= (Patient OR Patients OR hospitalized OR hospitalised OR admitted) )  *AND* LANGUAGE:  (English)  *Indexes=SCI-EXPANDED, SSCI, A&HCI, ESCI Timespan=2019-2021* | [1,093,510](http://apps.webofknowledge.com/summary.do?product=WOS&doc=1&qid=2&SID=F5blfoj97UWodAibDV3&search_mode=AdvancedSearch&update_back2search_link_param=yes) |
| #1 | (TS=(“Wuhan coronavirus” OR “COVID19*” OR “COVID-19*” OR “COVID-2019*” OR “coronavirus disease 2019” OR “SARS-CoV-2” OR “2019-nCoV” OR “2019 novel coronavirus” OR “severe acute respiratory syndrome coronavirus 2” OR “2019 novel coronavirus infection” OR “coronavirus disease 2019” OR “coronavirus disease-19” OR “SARS-CoV-2019” OR “SARS-CoV-19”) )  *AND* LANGUAGE:  (English)  *Indexes=SCI-EXPANDED, SSCI, A&HCI, ESCI Timespan=2019-2021* | [124,156](http://apps.webofknowledge.com/summary.do?product=WOS&doc=1&qid=1&SID=F5blfoj97UWodAibDV3&search_mode=AdvancedSearch&update_back2search_link_param=yes) |
| **SCOPUS SEARCH STRATEGY 1 JUNE 2021** | | |
| #1 | TITLE-ABS-KEY ("Wuhan coronavirus" OR "Wuhan seafood market pneumonia virus" OR "COVID19*" OR "COVID-19*" OR "COVID-2019*" OR "coronavirus disease 2019" OR "SARS-CoV-2" OR sars2 OR "2019-nCoV" OR "2019 novel coronavirus" OR "severe acute respiratory syndrome coronavirus 2" OR "2019 novel coronavirus infection" OR "coronavirus disease 2019" OR "coronavirus disease-19" OR "novel coronavirus" OR coronavirus OR "SARS-CoV-2019" OR "SARS-CoV-19") AND TITLE-ABS-KEY (Patient OR Patients OR hospitalized OR hospitalised OR "hospitalized patients" OR "hospitalized patients") AND TITLE-ABS-KEY ( "nosocomial infection" OR "cross infection" OR "hospital acquired infection" OR "healthcare associated infection" OR "healthcare-associated infections" OR "healthcare-acquired infections" OR "hospital acquired infections" OR nosocomial OR "healthcare associated" OR "hospital acquired" OR "hospital-acquired" OR "healthcare-associated" OR "healthcare spread" OR "healthcare case" OR "healthcare incidence") AND ( LIMIT-TO ( PUBYEAR,2021) OR LIMIT-TO ( PUBYEAR,2020) OR LIMIT-TO ( PUBYEAR,2019) ) | 1 303 |

The detailed search terms used for the search strategies in PubMed, Scopus and Web of Science. These strategies were finalized after several re-iterations testing different combinations of search terms to obtain an exhaustive search output. Total articles yielded from the final search is shown.
